# Atypical echocardiographic phenotype of end-stage hypertrophic cardiomyopathy: a case series

**DOI:** 10.3389/fcvm.2026.1907550

**Published:** 2026-10-09

**Authors:** Xi Wang, Qiuying Liu, Li Zhao, Menglu Li

**Affiliations:** 1 Department of Ultrasonography, Sixth Medical Center of PLA General Hospital, Beijing, China; 2 Department of Cardiology, Sixth Medical Center of PLA General Hospital, Beijing, China; 3 Department of Radiology, Sixth Medical Center of PLA General Hospital, Beijing, China

**Keywords:** cardiac arrest, case series, echocardiography, end-stage, hypertrophic cardiomyopathy

## Abstract

We fully documented the diagnostic process of three hypertrophic cardiomyopathy (HCM) cases without typical asymmetric myocardial hypertrophy, including medical histories, imaging, and laboratory tests, ultimately confirming the diagnosis through genetic testing. Case 1 presented with segmental wall motion abnormalities with thrombosis, requiring differentiation from coronary atherosclerotic heart disease; case 2 presented with restrictive hemodynamic changes that could be confused with restrictive cardiomyopathy such as amyloidosis; case 3 occurred in a pediatric setting and showed diffuse and concentric hypertrophy similar to metabolic disorders. Various imaging examinations were employed for differential diagnosis, and whole-exome sequencing identified a pathogenic MYBPC3 mutation in case 1 and pathogenic MYH7 mutations in cases 2 and 3, including a *de novo* variant in case 3. We provided appropriate treatment and follow-up for each case. Case 1's left ventricular ejection fraction increased to 48%, and cases 2 and 3 have not experienced worsening heart failure or malignant arrhythmic events based on an optimized drug regimen. This case series confirms the diversity of echocardiographic phenotypes in end-stage HCM, underscoring the role of multimodal imaging and genetic testing in diagnosis and differential diagnosis.

## Introduction

Approximately 70% of hypertrophic cardiomyopathy (HCM) cases are attributed to pathogenic variants in the *β*-myosin heavy chain (MYH7) and myosin-binding protein C (MYBPC3) genes (1). Nevertheless, the clinical phenotypes are highly heterogeneous. Typical manifestations of HCM include asymmetric left ventricular wall hypertrophy, systolic anterior motion of the mitral valve, and left ventricular outflow tract obstruction. End-stage HCM is defined by a left ventricular ejection fraction (LVEF) <50% and carries a high annual mortality rate of up to 13.2% (2). In this stage, the loss of typical non-uniform myocardial hypertrophy results in complex and heterogeneous echocardiographic manifestations, which often lead to delayed diagnosis and misdiagnosis. In the present study, we present the diagnostic process of three cases of end-stage HCM with atypical echocardiographic features.

## Case report

### Case 1

#### Chief complaints and clinical findings

A 50-year-old male presented to the cardiology clinic with occasional syncope for 1 year. He had incidental findings of left heart thickening for 20 years, and a history of palpitations after physical labor for 10 years. He had a family history of hereditary disease (his mother and second son had been diagnosed with HCM, both alive). Physical examination: Bilateral lung percussion was resonant, with clear breath sounds, without rales or friction rubs. There was no precordial bulge, heave, or thrills; normal cardiac dullness borders were normal. The patient had a regular heart rate of 54 bpm. Blood pressure was 106/66 mmHg. There was no jugular venous distension, periorbital or lower-limb edema, and a systolic murmur was heard at the third to fourth left midclavicular intercostal spaces. New York Heart Association (NYHA) functional classification was II. Laboratory tests: N-terminal pro-B-type Natriuretic Peptide (pro-BNP) 669 ng/L; troponin I <0.01 ng/mL. The dynamic electrocardiogram showed ventricular premature beats (VPB); ST-segment depression in III, aVF, and V6; and flattened or biphasic T waves in III, aVF, and V5–V6.

#### Echocardiography

Echocardiography (see Supplementary Table 1 for details**)** showed myocardial thinning (5 mm) and segmental abnormal ventricular wall motion in the interventricular septum and left ventricular anterior segment, local myocardial echo enhancement (fibrosis manifestation) accompanied by thrombus. Localized thickening of the basal segment of the interventricular septum (21 mm) was present, with an isovolumetric relaxation time (IVRT): 42 ms, left atrial volume index (LAVI): 43.9 mL/m^2^. Diastolic dysfunction grading was Grade II according to the 2016 ASE/EACVI recommendations (3). LVEF: 40%.

#### Differential diagnosis

Segmental wall motion abnormalities, myocardial fibrosis, intracardiac thrombus, and ECG ST-T wave abnormalities strongly suggested coronary atherosclerotic heart disease and myocardial infarction. However, no coronary artery stenosis was found 4 years ago on coronary angiography at another hospital, and the patient had not experienced chest pain since then. Coronary magnetic resonance angiography (MRA) at the current admission revealed patent coronary ostia. Given the low likelihood of significant coronary stenosis developing within 4 years without traditional risk factors (hypertension, diabetes, smoking, etc.), coronary artery disease was essentially ruled out, and repeat invasive coronary angiography was not planned. Contrast cardiac magnetic resonance (CMR) demonstrated extensive patchy late gadolinium enhancement (LGE) within the left ventricle (LGE burden = 54.1%), consistent with HCM-induced myocardial fibrosis rather than an ischemic origin. Genetic testing (whole-exome sequencing) revealed a pathogenic MYBPC3 mutation, leading to the final diagnosis of end-stage HCM (hypodynamic dilated phenotype); see Supplementary Table 2 for details. Imaging and genetic findings are shown in Figure 1.

**Figure 1:**
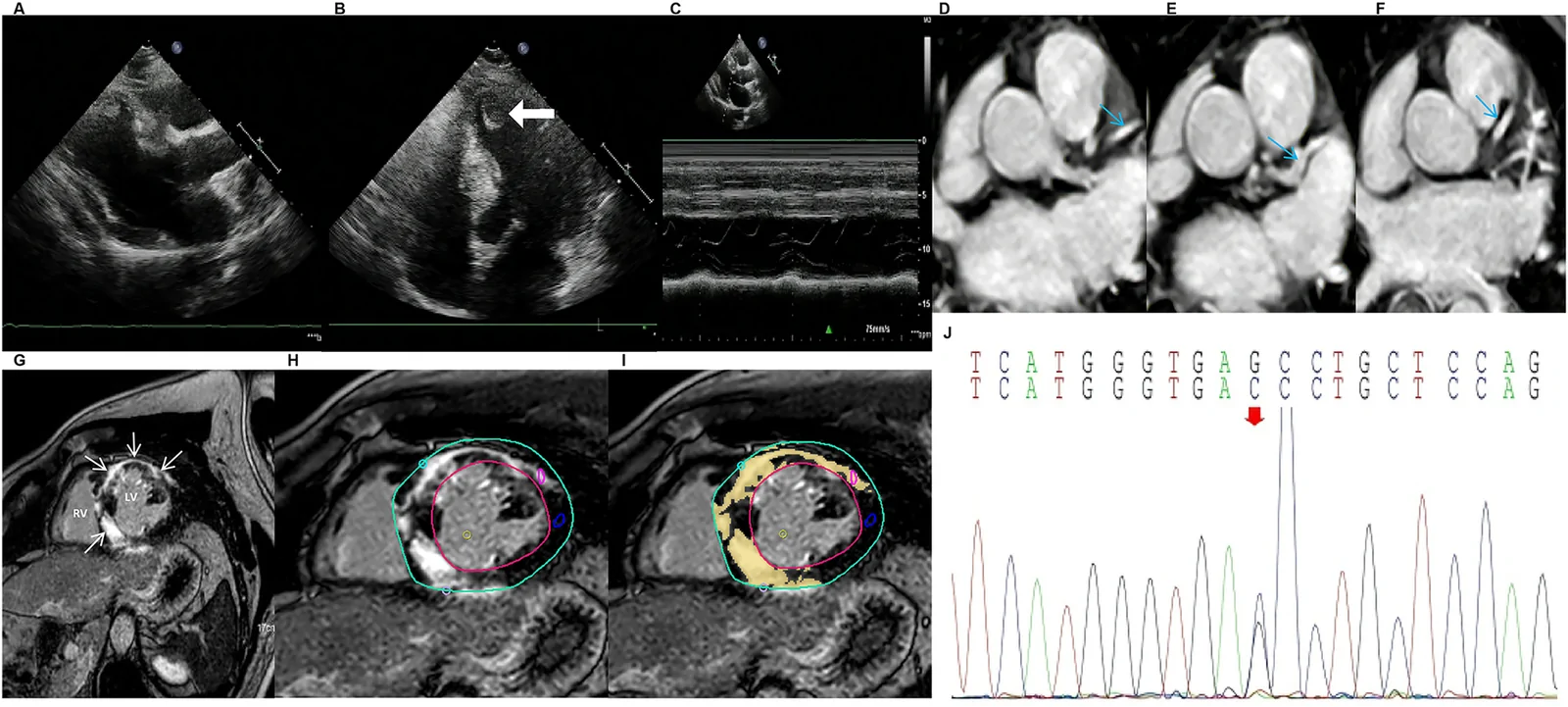
Multimodal imaging findings and genetic testing of case 1. Echocardiography parasternal long-axis view **(A)** shows thinning of the interventricular septum in the middle segment (5 mm) and thickening of the basal segment (21 mm). Four-chamber apical view **(B)** shows apical thrombus (arrow mark). Parasternal long-axis Chordae Level M-mode **(C)** shows markedly reduction in interventricular septal motion. Coronary MRA **(D–F)** shows patent coronary ostia. (arrow mark). CMR Biventricular Short-Axis View **(G)** shows extensive patchy enhancement of the left ventricle (arrow mark). LGE-CMR **(H,I)** showing extensive myocardial fibrosis. Pink region of interest (ROI): enhanced area reference; blue ROI: normal myocardial reference; yellow area: automatically segmented LGE regions. Genetic testing **(J)** indicates a splice site mutation in the MYBPC3 gene at position chr11: 47362684 on chromosome 11. The guanine base in the fifth intron downstream of nucleotide 1897 has been replaced by a cytosine.

#### Therapeutic interventions

A left ventricular apical aneurysm was identified on CMR in this patient, representing a key predisposing factor for left ventricular thrombus formation (4). According to current expert consensus, primary prophylactic anticoagulation should be considered for this case (5). Anticoagulation with rivaroxaban was promptly initiated since the definitive diagnosis of apical thrombosis was established. Following an in-hospital multidisciplinary team (MDT) discussion, lifelong anticoagulation as a secondary prevention strategy was deemed necessary for this patient with end-stage hypertrophic cardiomyopathy complicated by left ventricular thrombus. Sacubitril/valsartan was prescribed to mitigate adverse myocardial remodeling. Based on the 2020 AHA/ACC risk stratification criteria for sudden cardiac death (SCD), the patient harbored three major risk factors: unexplained syncope, LVEF <50%, and LGE burden >15% (Supplementary Table 3). Given this high-risk profile, we recommended implantable cardioverter defibrillator (ICD) implantation under a Class IIa indication (Level of Evidence: B-NR) (6), which, regrettably, was refused by the patient for economic reasons.

#### Follow-up and outcomes

At the 3-month follow-up, echocardiography showed resolution of the thrombus, and the LVEF increased to 48%. Six months following hospital discharge, there was no recurrence of syncope in the patient. The clinical course and timeline of this case are summarized in Table 1.

**Table 1:** Timeline of the three cases.

Time point	Case 1	Case 2	Case 3
Past history	20 years earlier: Echo check showed heart thickening. 10 years earlier: Palpitations after exertion. 4 years earlier: coronary angiography showed no stenosis	7 days before admission: new-onset hypertension	Age 7: diagnosed with LV hypertrophy (15 mm); CMR with LGE; genetic testing identified *de novo* MYH7 pathogenic variant
Onset and admission	Presented with exertional syncope for 1 year	Presented with exertional chest tightness and dyspnea for 2 months	2 months prior: out-of-hospital cardiac arrest; received cardiopulmonary resuscitation (CPR) and rehabilitation
Key examinations	ECG: ventricular premature complex (VPC) and ST-T abnormalities; Echo: segmental wall motion abnormality, apical thrombus, LVEF 40%; CMR: extensive LGE	ECG: atrial fibrillation, NSVT, ST-T changes; Echo: biatrial enlargement, restrictive filling, LVEF 44%; ^99m^Tc-PYP: negative	Echo: diffuse concentric hypertrophy, GLS −9%, LVEF 49%; CMR: LVEF 46%; pro-BNP and troponin I markedly elevated
Genetic diagnosis	MYBPC3 c.1897 + 5G > C (likely pathogenic)	MYH7 c.1544T > G (pathogenic)	MYH7 c.2155C > T (pathogenic, *de novo*)
Treatment	Rivaroxaban; sacubitril/valsartan	Rivaroxaban; diuretics; dapagliflozin; *β*-blocker; valsartan	Rehabilitation; ICD implantation recommended strongly
Latest follow-up	3-month follow-up: thrombus resolved, without recurrent syncope	Asymptomatic at discharge; planned for follow-up echocardiography	Conscious, independent sitting, assisted walking; under regular follow-up

#### Patient perspective

The patient showed good compliance with the prescribed treatment regimens. He and his family lived with constant worry about sudden cardiac death risk and the possibility of disease passing to other family members. Their concerns intensified after the patient's second son received an HCM diagnosis and began mavacamten treatment, prompting ongoing unease about long-term cardiac prognosis and genetic screening for other relatives.

### Case 2

#### Chief complaints and clinical findings

A 51-year-old man presenting with chest tightness and shortness of breath on exertion for 2 months presented to the cardiology clinic. He had no family history of cardiovascular disease. On physical examination, bilateral lung percussion was resonant with clear breath sounds, free of rales or pleural rubs. Cardiac exam revealed no precordial bulge, heaves, or thrills, normal cardiac dullness borders, a regular heart rate of 102 beats/min, and no pathological murmurs or pericardial rubs at the valve auscultation sites. No jugular distension, abnormal venous pulsation, periorbital, or lower-limb edema was observed. His blood pressure was previously normal and had been elevated for 7 days, reaching a maximum of 160/130 mmHg. The NYHA functional classification was III. Laboratory tests demonstrated an N-terminal pro-BNP level of 1,620 ng/L; and a troponin I <0.01 ng/mL. Ambulatory electrocardiogram showed atrial fibrillation, non-sustained ventricular tachycardia (NSVT), ST-segment depression in leads V5–V6, inversion and biphasic T waves in I–III, aVF, aVL, and V4–V6.

#### Echocardiography

After the blood pressure was controlled with oral antihypertensive medications, transthoracic echocardiography was performed. Echocardiography (Supplementary Table 1**)** showed uniform left ventricular wall thickening (17 mm) and significant enlargement of both atria (LAVI 53.8 mL/m^2^). Spectral Doppler revealed a single-peak E wave at the mitral valve orifice in the diastolic period, and a markedly elevated ratio between early mitral inflow velocity and mitral annular early diastolic velocity (E/e’), septal E/e’:16.7, DT: 70 ms. Systolic pulmonary arterial pressure was 40 mmHg, assessed by tricuspid regurgitation. According to the 2016 ASE/EACVI recommendations (3), these findings indicated elevated mean left atrial pressure and left ventricular diastolic dysfunction, and the LVEF was 44%.

#### Differential diagnosis

The ultrasound findings were similar to restrictive cardiomyopathy, particularly cardiac amyloidosis. The relevant tests had been scheduled. Serum and urine immunofixation electrophoresis revealed no monoclonal bands. All assays for kappa and lambda light chains, as well as IgG, IgA, and IgM heavy chains, yielded negative results. Serum total kappa and lambda light chains were 9.53 and 4.67 g/L, respectively, yielding a *κ*/*λ* ratio of approximately 2.04. Notably, these measurements represent total immunoglobulin light chains rather than serum free light chains (sFLC), and dedicated sFLC testing was not performed in this patient. No laboratory findings demonstrated clonal plasma cell dyscrasia, making light-chain amyloidosis less likely; ^99m^Tc-PYP myocardial scintigraphy with 1- and 3-h delayed imaging showed a heart-to-contralateral-lung (H/CL) ratio of 1.29 at 1 h and 1.17 at 3 h (both below the diagnostic cutoff value of ≥1.5). No focal myocardial tracer retention was observed throughout acquisition. Visual uptake was graded Perugini Grade 0, with no abnormal myocardial radiotracer accumulation and equivalent radioactivity between the myocardium and intracardiac blood pool. Transthyretin-related amyloidosis was excluded based on standardized nuclear interpretative criteria. CMR revealed patchy enhancement at the apex of the heart (LGE burden = 26.1%), which is inconsistent with the diffuse myocardial involvement typical of amyloidosis. Genetic testing (whole-exome sequencing; see Supplementary Table 2 for details) revealed a pathogenic MYH7 mutation, confirming the final diagnosis of end-stage HCM (hypodynamic restrictive phenotype). Imaging and genetic findings are shown in Figure 2.

**Figure 2:**
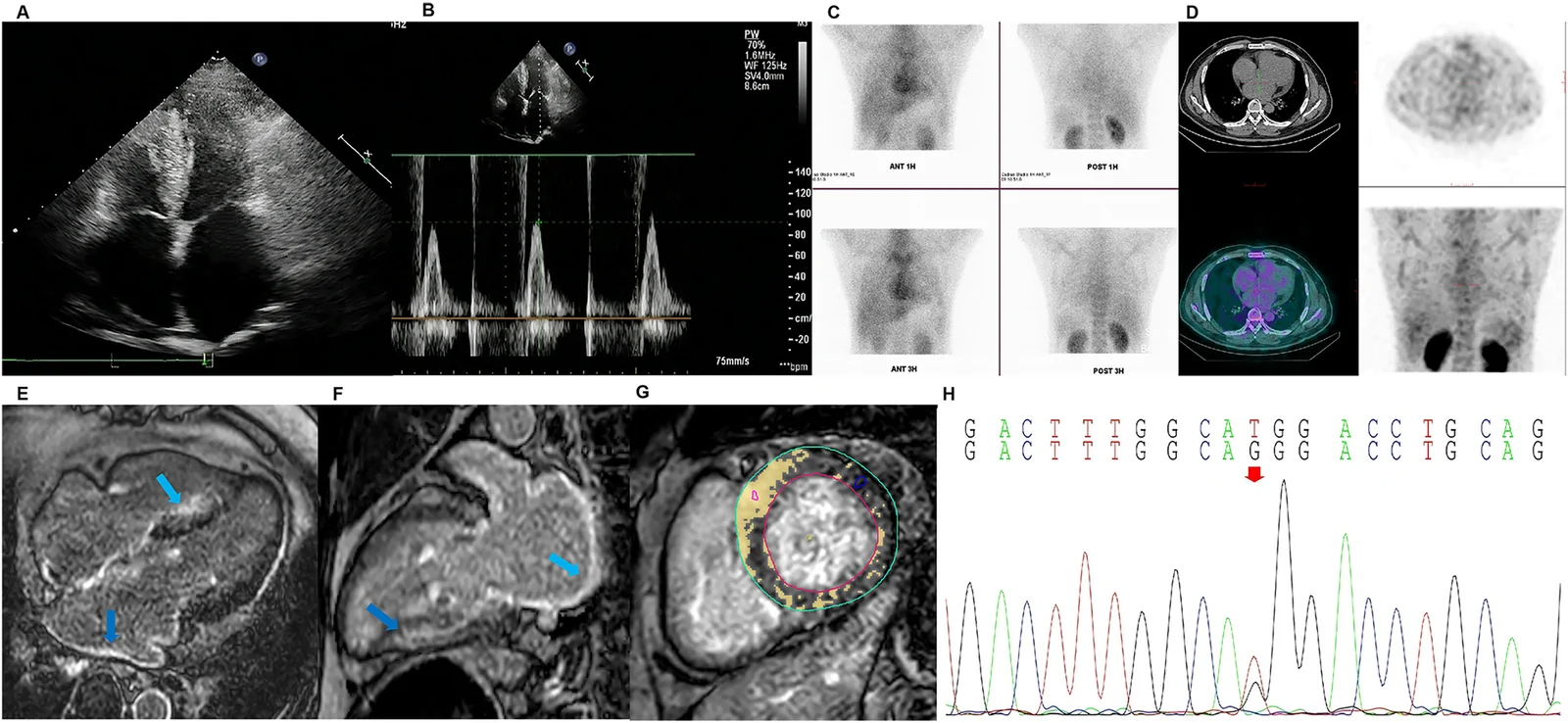
Multimodal imaging findings and genetic testing of case 2. Echocardiography **(A)** shows significant enlargement of both atria. Spectral Doppler **(B)** shows mitral valve spectrum with peaked E wave. ^99m^Tc-PYP scintigraphy **(C,D)** demonstrates absent myocardial radiotracer uptake, with equivalent radioactivity between the myocardium and intracardiac blood pool. LGE-CMR acquired in four-chamber **(E)** and two-chamber **(F)** planes reveals focal hyperenhancement of the interventricular septal myocardium (pale blue arrow) and pathological atrial wall enhancement (dark blue arrow). Short-axis apical biventricular LGE-CMR **(G)** shows extensive transmural myocardial fibrosis. ROIs are annotated as follows: pink, region of pathological enhancement; green, normal reference myocardium; yellow, automatically segmented LGE-positive myocardium. Genetic sequencing **(H)** identifies a pathogenic missense variant in MYH7 (chr14:23897743), corresponding to a c.1544T > G nucleotide substitution.

#### Therapeutic interventions

The patient was prescribed oral rivaroxaban for anticoagulation due to atrial fibrillation. During hospitalization, furosemide, spironolactone, and dapagliflozin were administered to alleviate diastolic heart failure; valsartan was used to attenuate myocardial remodeling, and metoprolol succinate was prescribed for heart rate control. The patient had three major high-risk markers: non-sustained ventricular tachycardia, LVEF <50%, and an LGE burden >15% (Supplementary Table 3). These findings placed the patient in the high-risk category, so we recommended ICD implantation under a Class IIa indication (Level of Evidence: B-NR) (6), which was declined by the patient. The clinical course and timeline of this case were summarized in Table 1.

#### Follow-up and outcomes

He was followed up by phone six months after discharge. He remained free of chest tightness and other discomforts, without subsequent hospital readmission.

#### Patient perspective

After receiving standardized medical treatment, the patient felt content with his physical condition and maintained a positive attitude toward disease self-management. From his personal standpoint, he saw no need for hospital readmission or ICD implantation.

### Case 3

#### Chief complaints and clinical findings

A 13-year-old girl was admitted to the pediatric department of our hospital for rehabilitation intervention of hypoxic-ischemic encephalopathy. She had no family history of cardiovascular disease. On physical examination, breath sounds were coarse bilaterally without rales or rubs, with a regular sinus rhythm, no cardiac murmurs, no jugular venous distension, or leg edema. The patient was alert with intact eye tracking, preserved comprehension but hypophonia and dysarthria, and normal palatal, ocular, and pupillary light reflexes. She was bedridden, able to roll over and lift her lower limbs yet clumsy limb movement, unable to sit upright; limb weakness and intermittent hypertonia, ataxia unassessable due to poor cooperation. Superficial and deep sensation were grossly intact. The corneal reflex was normal, abdominal reflexes were blunted, knee jerks were absent. Functional grade: NYHA Class I. Laboratory tests: pro-BNP level of 407 ng/L. Ambulatory electrocardiogram showed ST-segment depression in leads II, III, aVF, V5, and V6; inverted T waves in leads III, aVF, V3, and V6. CMR imaging demonstrated an LVEF of the 46%, and the global longitudinal strain (GLS) of the left ventricle was －7.9%.

Past medical history: At the age of 7 years (6 years prior to admission), she was diagnosed with HCM at another hospital. At that time, the maximal thickness of the left ventricular wall was 15 mm, with an LVEF of 71%; and CMR detected LGE. Whole-exome sequencing performed concurrently identified a pathogenic variant in the MYH7 gene associated with HCM, whereas no variant was detected at the same locus in either parent. She suffered sudden cardiac arrest and was taken to a children’ s hospital 2 months ago; pro-BNP: 2,629 ng/L; troponin I: 12.37 ng/mL. Echocardiography showed left ventricular hypertrophy and a borderline normal LVEF of 51%. After cardiopulmonary resuscitation, she had significant motor function impairment.

#### Echocardiography

Echocardiography showed diffuse and concentric hypertrophy of the left ventricle, with a maximum wall thickness of 21 mm, enhanced myocardial echo with coarsened texture, LAVI: 33 mL/m^2^. The mitral inflow spectrum demonstrated an L wave, which was an indicator of diastolic dysfunction. Diastolic dysfunction grading was Grade I (3) and the LVEF 49%. The global longitudinal strain of the left ventricle was markedly decreased to －9%. See Supplementary Table 1 for details.

#### Differential diagnosis

Diffuse concentric myocardial hypertrophy with onset in childhood and no family history raised suspicion of secondary hypertrophy from metabolic/endocrine disorders or lysosomal storage diseases. The Milslab MZ01 metabolic screening panel (Panel A: plasma acylcarnitine profiling; Panel B: urinary amino acids; Panel C: urinary organic acids and special metabolites) was performed to exclude metabolic phenocopies of hypertrophic cardiomyopathy, such as fatty acid oxidation defects, organic acidurias, urea cycle abnormalities, and mitochondrial energy metabolism disorders. No abnormal metabolic biomarkers were identified in this patient, excluding metabolic cardiomyopathies caused by mass spectrometry. This assay cannot diagnose lysosomal or glycogen storage cardiomyopathies [Pompe disease (GAA), Danon disease (LAMP2), Fabry disease (GLA), PRKAG2 syndrome(PRKAG2)], which require enzymatic testing or targeted gene sequencing for confirmation. CMR was acquired on a Philips Ingenia 1.5 T scanner. The normal native T1 reference threshold at our center is approximately 1,000 ms. Case 3 exhibited a native T1 value of 1,079 ms without T1 shortening. Decreased native T1 represents the characteristic imaging hallmark of Anderson–Fabry disease. The normal T1 value in this patient supports the exclusion of storage cardiomyopathy (7). Coarsened, heterogeneous myocardial echotexture was inconsistent with the uniform, vacuolar myocardial changes characteristic of storage disease, and whole-exome sequencing identified a pathogenic MYH7 mutation with wild-type genotypes at the corresponding loci in parental genetic testing (see Supplementary Table 2 for details), which led to the diagnosis of sporadic HCM (pediatric-onset, concentric hypertrophic phenotype). Imaging and genetic findings are shown in Figure 3. Furthermore, a retrospective review of the 6-year disease course in this case revealed the following key findings: At age 7, the left ventricular wall thickness was 15 mm, and myocardial LGE was already evident on CMR. A series of tests during this hospitalization supported myocardial dysfunction, such as elevated BNP, an LVEF of 46% on CMR, an LVEF of 49% on echocardiography, and a markedly reduced GLS of −9% (8). Based on these findings, a diagnosis of end-stage HCM was confirmed.

**Figure 3:**
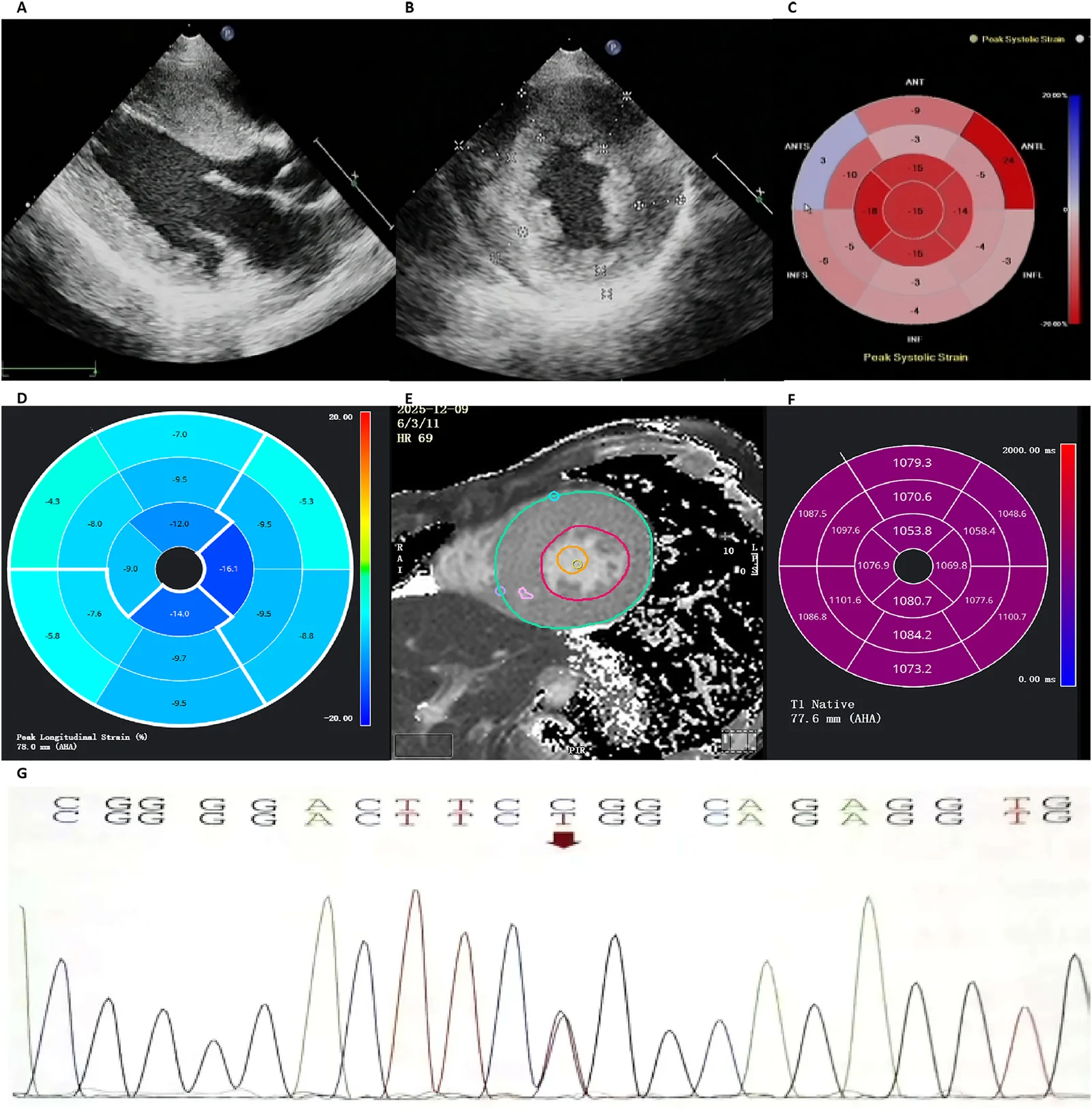
Multimodal imaging findings and genetic testing of case 3. Transthoracic echocardiography demonstrates concentric, diffuse myocardial hypertrophy with increased myocardial echogenicity (**A**,**B**). Speckle-tracking imaging yields a left ventricular GLS of −9.0% **(C)**; CMR-derived global longitudinal strain **(D)** is −7.9%. Native T1 mapping CMR **(E,F)**: the global native myocardial T1 value averages 1,079 ms, with a slice-specific mean myocardial native T1 of 1,081 ms, blood-pool native T1 of 1,522 ms, and focal pathological myocardium within the pink ROI measuring 1,257 ms. Genetic sequencing **(G)** identifies a pathogenic missense variant in MYH7 (chr14:23895180), corresponding to a c.2155C > T nucleotide substitution.

#### Therapeutic interventions

Case 3 was a 13-year-old pediatric patient. Given that the HCM Risk-SCD model (4) is validated only for patients older than 16 years, the Precision Medicine for Cardiomyopathy (PRIMaCY) score was applied for risk stratification in this child (available online at https://www.mdcalc.com/calc/10438/precision-medicine-cardiomyopathy-primacy-score), which yielded an adjusted 5-year SCD risk of 95.4%, indicating high risk. Furthermore, owing to the prior history of cardiopulmonary arrest, ICD implantation was strongly recommended for secondary prevention according to the 2024 AHA/ACC HCM guidelines (6) (Class I recommendation, Level of Evidence: B-NR). However, this intervention was declined by the patient's guardians, and they focused only on neurological rehabilitation therapy. Furthermore, oral administration of the *β*-blocker metoprolol was initiated for hypertrophic cardiomyopathy.

#### Follow-up and outcomes

At the 6-month follow-up, the patient demonstrated a good response to neurological rehabilitation. She exhibited clear consciousness, with normal muscle tone and mild reduction in muscle strength. She was able to sit independently, stand unassisted, and walk with support. Furthermore, beta-blocker (metoprolol) was maintained postdischarge. The clinical course and timeline of this case are summarized in Table 1.

#### Patient perspective

Their daughter’s cardiac arrest brought severe psychological burden to the couple, who endured constant fear and doubt regarding the child’s long-term cardiac and neurological prognosis. With multidisciplinary counseling, their anxiety gradually eased, and they fully understood the disease management plan from their own perspective. Although they remained hesitant to proceed with ICD implantation, they sincerely appreciated the medical team’s care and promised to adhere to regular follow-up visits.

## Discussion

This case series highlights the phenotypic heterogeneity and diagnostic challenges of end-stage HCM, supporting the integration of detailed clinical and family histories with multimodality imaging and molecular genetic testing for accurate diagnosis.

The morphological and functional manifestations of end-stage HCM span two extremes (9). The first is the hypodynamic dilated phenotype, characterized by increased left ventricular volume and spherical remodeling, leading to the transfer of left heart pressure to the right heart, which directly affects the basic elements of the Venturi effect, resulting in Systolic Anterior Motion (SAM) regression and the disappearance of left ventricular outflow tract obstruction (10). At this stage, structural changes occur in the middle layer of the intramural coronary vasculature, resulting in an imbalance between oxygen supply and demand in the coronary microcirculation and myocardial mass. Myocardial ischemia occurs in both hypertrophic and non-hypertrophic areas, leading to cardiomyocyte death and replacement with focal or transmural scars. Ventricular wall motion disorders provide a pathological basis for intraventricular thrombus formation (11, 12). Case 1 in the present study falls into this category and requires differentiation from coronary artery disease. Twenty-year disease progression in this patient featured myocardial transformation from hypertrophy to thinning with diffuse fibrosis and progressive ventricular dilatation, culminating in impaired cardiac function.

The second is the hypodynamic restrictive type, often accompanied by atrial fibrillation, characterized by diastolic dysfunction and significant atrial dilation, similar to restrictive cardiomyopathy. During HCM, myocardial ischemia and hypoxia impair its active relaxation ability, while fibrosis leads to stiff movement and limited passive filling. In addition, atrial fibrillation affects effective atrial contraction, making it insufficient to compensate for the decreased diastolic function. The elevated left ventricular end-diastolic pressure leads to an abrupt reduction in filling volume, while disordered myocardial electrical activity promotes left ventricular fibrosis, eventually forming a vicious cycle of progressive deterioration in diastolic function (13, 14). This type, just like case 2 in this study, requires exclusion of restrictive cardiomyopathy such as myocardial amyloidosis, and myocardial biopsy may be required if necessary. In addition, case 2 was newly diagnosed with hypertension 1 week prior to presentation. It is well recognized that hypertension-induced myocardial hypertrophy rarely exceeds 15 mm in thickness, typically presenting as eccentric hypertrophy accompanied by left ventricular cavity enlargement. In contrast, this patient had a very short duration of hypertension but exhibited a maximal left ventricular wall thickness of 17 mm, characterized by concentric hypertrophy and small left ventricular cavity dimension. Furthermore, no left ventricular high voltage was noted on electrocardiogram, which is a typical hallmark of hypertensive heart disease. Taken together, hypertension could be excluded as the primary etiology of myocardial hypertrophy in this patient. A special phenotype of HCM and adverse events rarely occur before puberty (15). Case 3 occurs in a child who completely differs from typical HCM onset in youth and middle age, which led us to go down the wrong diagnostic direction at first. Concentric hypertrophy is atypical and easily misdiagnosed as “myocardial hypertrophy” associated with storage diseases. However, the pathological basis of the latter is lysosomal glycogen accumulation, significant swelling of the cytoplasm filled with “vacuolar” structures, with uniform myocardial thickening and echo enhancement. In contrast, in most patients with HCM, the massive, disordered arrangement of the collagen matrix increases ultrasonic reflection interfaces within the myocardium, which results in global myocardial echogenicity enhancement with a relatively diffuse, uneven distribution pattern (16–18). The present case could easily be misdiagnosed based on echocardiographic features alone.

Genetic testing is key to the final diagnosis. Two heterozygous missense variants in MYH7 were identified in case 2 (p.Met515Arg, c.1544T > G) and case 3 (p.Arg719Trp, c.2155C > T); both localize to the S1 head domain of *β*-myosin heavy chain. Specifically, p.Met515Arg resides within the actin-binding subdomain of the S1 head, while p.Arg719Trp is located in the converter subdomain of the S1 head. Both variants are non-conservative mutations that alter amino acid charge and have been validated as malignant HCM-causative variants in large clinical cohorts (PMID: 15358028, PMID: 8282798), and are classified as Pathogenic per American College of Medical Genetics and Genomics (ACMG) criteria (19, 20).

Although p.Met515Arg and p.Arg719Trp interfere with distinct molecular interfaces, both disrupt the autoinhibited interacting heads motif (IHM) conformation and trigger hypercontractility at the myocardial molecular level. The p.Met515Arg variant lies in the Mesa region; this charge-altering mutation weakens binding between the S1 domain, proximal S2 segment, and myosin-binding protein C (MyBP-C), increasing the pool of functionally active myosin heads (Na) and upregulating actin-activated ATPase activity. By contrast, the R719Trp mutation impairs the head-head interaction interface, disrupting intramolecular interactions required to sequester myosin in an inactive state. This releases abundant activated myosin heads, ultimately increasing the population of force-generating cross-bridges within the sarcomere and driving the pathogenesis of HCM (21, 22).

Case 1 harbored a private rare intronic variant in MYBPC3 NM_000256.3:c.1897 + 5G > C, which does not directly alter the amino acid sequence. This variant is absent from ClinVar, gnomAD, and LOVD. No clinical cohort or *in vitro* functional studies have been published for this exact substitution. Though it induces no direct amino acid change at the DNA level, the SpliceAI score of 0.79 (cutoff ≥0.5 for strong splice-disrupting evidence) (23) predicts that the variant may disrupt canonical 5’ splice donor recognition of pre-mRNA.

While direct functional validation of this variant is lacking, prior studies have demonstrated that the +1 to +6 intronic region of 5′ splice donors constitutes a highly conserved core motif; G/C single nucleotide substitutions within this region abrogate normal splice signal recognition, resulting in MYBPC3 haploinsufficiency and subsequent hypertrophic myocardial remodeling (24, 25). Singer et al. confirmed via MYBPC3 cohort data and *in vitro* functional tests that the position-matched variant NM_000256.3:c.654 + 5G > C, carrying the same G > C substitution, abolishes canonical splicing and yields truncated abnormal transcripts (26). Based on conserved splice motifs, c.1897 + 5G > C is predicted to possess comparable pathogenicity, and its ACMG classification is likely pathogenic.

Pathogenic variants causing HCM follow an autosomal dominant inheritance pattern, conferring a 50% transmission risk to the offspring of probands (27). The disease exhibits incomplete penetrance; some variant carriers remain free of myocardial hypertrophy. Compared with MYH7 variants, mutations in MYBPC3 demonstrate lower penetrance, and carriers typically develop symptoms at an older age (28). Moreover, substantial phenotypic heterogeneity exists both within and across families, and sarcomeric mutations do not solely determine the clinical manifestations of HCM (1).

Beyond vertical familial transmission, *de novo* pathogenic variants account for sporadic cases without a positive family history. A negative family history cannot rule out genetic HCM, as incomplete penetrance, late-onset disease, or *de novo* mutations may mask genetic susceptibility. Among pediatric patients with HCM, *de novo* sarcomeric variants predict a higher risk of adverse cardiovascular events, which constitutes a critical molecular basis for the early-onset and severe clinical presentation seen in this young patient (29). Case 3 in our study harbored a *de novo* missense variant p.Arg719Trp in MYH7. This variant is associated with a markedly elevated risk of premature death (20) and increased susceptibility to severe complications including atrial fibrillation, heart failure, and SCD (30).

SCD is one of the most devastating complications and the leading cause of death in patients with HCM. Risk stratification for SCD, incorporating the European Society of Cardiology (ESC)-endorsed HCM Risk-SCD prediction model and the 2024 AHA/ACC HCM guidelines, to formulate targeted preventive and therapeutic strategies, constitutes a core component in the clinical management of patients with HCM. In this study, we utilized the 2020 AHA/ACC risk stratification algorithm to evaluate the risk of SCD among patients with HCM. Risk categorization often differs substantially between this algorithm and the ESC HCM Risk-SCD model. The HCM Risk-SCD calculator estimates the 5-year probability of SCD based on seven preset variables (31); however, it frequently underestimates risk in certain patient subgroups and omits several vital prognostic indicators, such as depressed left ventricular ejection fraction, extensive LGE, and left ventricular apical aneurysm. In comparison, the AHA/ACC guidelines list numerous independent SCD high-risk factors (6). Consequently, a subset of patients deemed at intermediate or low risk by the HCM Risk-SCD model still meet the high-risk thresholds laid out by the AHA/ACC criteria. This discrepancy leads to inconsistent clinical guidance for primary prevention with ICDs, particularly for individuals with end-stage HCM, diffuse myocardial fibrosis, or apical aneurysms. In addition, cohort studies of Chinese patients with HCM have verified that the AHA/ACC SCD risk stratification scheme outperforms the ESC HCM Risk-SCD model for SCD risk assessment (32). However, in this study, all three patients declined ICD implantation for distinct reasons. Case 1 declined ICD implantation owing to financial burden, with healthcare funds allocated to mavacamten treatment for his second son with HCM. Case 2 refused implantation as he experienced remarkable symptom relief after hospitalization and he deemed his exercise capacity increased and ICD implantation was unnecessary at present. While case 3's family recognized the value of ICD therapy, they intended to focus on rehabilitation first and reconsider device implantation later.

Notably, even when pathogenic sarcomeric variants are identified, systematic screening for phenocopies of HCM is mandatory. Such differential diagnoses include Fabry disease, Pompe disease, PRKAG2 syndrome, cardiac amyloidosis, and conditions induced by excessive pressure overload. These phenocopies differ fundamentally from sarcomeric HCM in genetic background, therapeutic regimens, and long-term prognosis; accordingly, standard SCD risk stratification tools for HCM cannot be directly applied to these patients.

## Limitation

First, all three patients declined ICD implantation, and no continuous ECG monitoring was conducted postdischarge. As a result, arrhythmia recurrence could not be identified except in the event of sudden cardiac death.

Second, the follow-up schedules were heterogeneous: although all three patients were followed up for 6 months after discharge, distinct follow-up outcomes were observed in each case. For Patient 1, left ventricular thrombus disappeared on echocardiography after 3 months of anticoagulant therapy. Patient 2 only completed telephone follow-up; the patient reported no obvious discomfort during daily 3-km jogging, yet no repeat cardiac imaging was performed. Physical examination of Patient 3 revealed satisfactory neurological rehabilitation. All three patients took medications strictly as instructed after discharge, and no recurrent cardiovascular symptoms such as chest tightness or chest pain were reported during follow-up.

## Conclusion

End-stage HCM can present with atypical echocardiographic phenotypes, posing significant challenges for diagnosis. This study presented three end-stage HCM cases with distinct echocardiographic features: multi-segmental wall motion abnormalities with intracardiac thrombus, restrictive hemodynamics with biatrial enlargement, and diffuse concentric left ventricular hypertrophy mimicking metabolic cardiomyopathy. All three patients were given individualized treatment. For patients with atypical HCM, comprehensive disease history procurement and multimodal imaging are crucial for differential diagnosis, and ultimately, genetic testing remains required for a definitive diagnosis.

## Data Availability

The data analyzed in this study are subject to the following licenses/restrictions: due to patient privacy protection regulations and institutional data security policies, the raw clinical datasets (including medical records, ultrasound and cardiac MRI data) cannot be made publicly available. All patient information has been fully anonymized in the manuscript. Qualified researchers may request access to the de-identified data from the corresponding author, subject to prior approval from the institutional ethics committee. Requests to access these datasets should be directed to Qiuying Liu (liuqiuying2014@163.com).
