# Supplementary material for: Atypical echocardiographic phenotype of end-stage hypertrophic cardiomyopathy: a case series
Source: Front Cardiovasc Med. 2026 Oct 9;13:1907550. doi: 10.3389/fcvm.2026.1907550 (PMC13690755; doi:10.3389/fcvm.2026.1907550)
Supplement: Supplementary file 1 [file Table1.docx]

Supplementary Table 1

Supplementary Table 1 **Echocardiographic characteristics and parameters of the 3 cases**

| **Echocardiographic parameter** | **Case 1** | **Case 2** | **Case 3** |
| --- | --- | --- | --- |
| Maximum thickness of the left ventricular wall (mm) | 21 | 17 | 21 |
| Ventricular wall thickening pattern | Local thickening of the basal segment of the interventricular septum | Uniform thickening of left ventricular wall | Diffuse and concentric hypertrophy of left ventricular wall |
| Left ventricular end-diastolic dimension (mm) | 58 | 43 | 35 |
| Left ventricular volume index (mL/m²) | 92 | 41 | 38 |
| Resting LVOTG (mmHg) | 3 | 3 | 5 |
| SAM of the mitral valve | （-） | （-） | （-） |
| Degree of mitral regurgitation | Mild | mild | mild |
| Left atrial anteroposterior diameter (mm) | 45 | 53 | 37 |
| Left atrial volume index (mL/m²) | 43.9 | 53.8 | 33 |
| Tricuspid regurgitation velocity (m/s) | 2.9 | 2.8 | 2.1 |
| PASP（mmHg） | 40 | 40 | 23 |
| Left ventricular ejection fraction | 40% | 44% | 49% |
| Global longitudinal strain of the left ventricle | -12% | -9% | -9% |
| E peak at mitral valve orifice (cm/s) | 28 | 92 | 32 |
| A peak at mitral valve orifice (cm/s) | 44 | / | 36 |
| E/A | 0.6 | / | 0.9 |
| Mitral deceleration time(ms) | 180 | 70 | 300 |
| Mitral isovolumic relaxation time(ms) | 42 | 60 | 90 |
| Septal e' (cm/s) | 3.8 | 5.5 | 4.9 |
| Lateral e' (cm/s) | 7 | 7.5 | 2.9 |
| Average E/e' | 5.2 | 14.1 | 8.2 |
| Diastolic dysfunction grading | II | II | I |
| Special signs | Multi-segmental motion abnormalities with thrombosis | Significant enlargement of both atria | Cardiac hypertrophy of left ventricular wall |
|  |  | Restrictive filling disorder |  |

Note: SAM: Systolic Anterior Motion;LVOTG: Left Ventricular Outflow Tract Pressure gradient;PASP: Pulmonary Artery Systolic Pressure
