# Supplementary material for: Atypical echocardiographic phenotype of end-stage hypertrophic cardiomyopathy: a case series
Source: Front Cardiovasc Med. 2026 Oct 9;13:1907550. doi: 10.3389/fcvm.2026.1907550 (PMC13690755; doi:10.3389/fcvm.2026.1907550)
Supplement: Supplementary file 2 [file Table2.docx]

Supplementary Table 2

**Supplementary Table 2.** **Genetic variant reporting of the 3 cases**

|  | **case 1** | **case 2** | **case 3** |
| --- | --- | --- | --- |
| Gene symbol | MYBPC3 | MYH7 | MYH7 |
| Variant | NM_000256.3:c.1897+5G>C | NM_000257.4:c.1544T>G（p.Met515Arg） | NM_000257.4:c.2155C>T（p.Arg719Trp） |
| ACMG classification | Likely pathogenic | Pathogenic | Pathogenic |
| Evidence | PS1、PP3_Moderate、PM2_Supporting | PP3_Strong、PM1、PM5、PS4_Supporting、PM2_Supporting、PP2 | PS1、PS2、PM1、PM2、PP2、PP3、PP5 |
| Protein consequence | No direct amino acid change | p.Met515Arg | p.Arg719Trp |
| Prediction scores | SpliceAI：0.79；REVEL、SIFT、Polyphen2、MutationTaster（-） | REVEL: 0.938; SpliceAI: - ; | SIFT：Deleterious; |
|  |  | SIFT：Deleterious; | PolyPhen2：Possibly damaging; |
|  |  | PolyPhen2：Possibly damaging; | MutationTaster：disease_causing_automatic |
|  |  | MutationTaster：disease causing; |  |
| ClinVar | No reported in ClinVar | VCV000407187.8 | VCV000014104.46 |
|  | Both father and second son were heterozygous for the same variant |  | Heterozygous de novo variant: |
|  |  |  | No variant detected in either parent at the same locus |
|  | No RNA/minigene studies performed |  |  |
