# Supplementary material for: Atypical echocardiographic phenotype of end-stage hypertrophic cardiomyopathy: a case series
Source: Front Cardiovasc Med. 2026 Oct 9;13:1907550. doi: 10.3389/fcvm.2026.1907550 (PMC13690755; doi:10.3389/fcvm.2026.1907550)
Supplement: Supplementary file 3 [file Table3.docx]

Supplementary Table 3

**Supplementary Table 3.** **SCD risk assessment indicators of the 3 cases**

| **Variable** | **Case1** | **Case2** | **Case3** |
| --- | --- | --- | --- |
| Age (years) | 50 | 51 | 13 |
| Maximum wall thickness (mm) | 21 | 17 | 21 |
| Left atrial diameter (mm) | 45 | 53 | 37 |
| LVOT gradient (mmHg) | 3 | 3 | 5 |
| Unexplained syncope | Present | Absent | Absent |
| NSVT | Absent | Present | Absent |
| Family history of SCD | Absent | Absent | Absent |
| LVEF | 40% | 44% | 49% (echocardiography)  46% (CMR) |
| History of cardiac arrest | Absent | Absent | Present |
| LGE | 54.1% | 26.0% | － |

Note: SCD: Sudden Cardiac Death;LVOTG: Left Ventricular Outflow Tract Pressure gradient;LVEF:Left Ventricular Ejection Fraction
